# Synergistic effects of dissolved organic carbon and inorganic nitrogen on methane uptake in forest soils without and with freezing treatment

**DOI:** 10.1038/srep32555

**Published:** 2016-08-30

**Authors:** Haohao Wu, Xingkai Xu, Cuntao Duan, Tuansheng Li, Weiguo Cheng

**Affiliations:** 1 State Key Laboratory of Atmospheric Boundary Layer Physics and Atmospheric Chemistry, Institute of Atmospheric Physics, Chinese Academy of Sciences, Beijing 100029, China; 2 Department of Atmospheric Chemistry and Environmental Science, College of Earth Science, University of Chinese Academy of Sciences, Beijing 100049, China; 3 College of Earth Science and Resources, Chang’an University, Xi’an 710054, China; 4 Faculty of Agriculture, Yamagata University, Tsuruoka 997-8555, Japan

## Abstract

There is limited knowledge about how the interaction of dissolved organic carbon (DOC) and inorganic nitrogen (N) released into the soil just after freezing can affect methane (CH_4_) uptake in forest soils. Here, we present how freezing treatment and glucose, as a DOC source, can affect the roles of NH_4_^+^-N and NO_3_^−^-N in inhibiting soil CH_4_ uptake, by using soil-core incubation experiments. A long-term freezing at low temperature reduced cumulative CH_4_ uptake in the soils sampled from two temperate forest stands without carbon (C) and N addition. The inhibition effects of N addition as NH_4_Cl and KNO_3_ on the soil CH_4_ uptake were much larger than C addition. Freezing treatment eliminated the inhibition effect of NH_4_Cl and KNO_3_ addition on CH_4_ uptake, and this response was affected by glucose addition and forest types. The addition of glucose eliminated the inhibition effect of NO_3_^−^-N on CH_4_ uptake in the forest soils without and with freezing treatment, while the addition of NH_4_^+^-N and glucose inhibited synergistically the soil CH_4_ uptake. The results highlight the importance of synergistic effects of DOC and N inputs on the soil CH_4_ uptake under forest stands during soil wetting and thawing periods.

Upland soils are regarded as the only biological sink of atmospheric methane (CH_4_) and are responsible for 6% of the global CH_4_ consumption^1^. Increasing atmospheric N deposition tends to inhibit the consumption of CH_4_ in upland soils, which may partly lead to the rising atmospheric abundance of CH_4_^2^,^3^. Ammonium (NH_4_^+^-N) and nitrate (NO_3_^−^-N) are regarded as main contributors of atmospheric wet N deposition, and the addition of NH_4_^+^-N to forest soils can suppress CH_4_ uptake^2^,^4^,^5^, while the effects of NO_3_^−^-N are contradictory with inhibition, no effect or stimulating effect on CH_4_ uptake^6^,^7^,^8^,^9^. Many researchers have reported that NO_3_^−^-N has a strong inhibitory effect on CH_4_ oxidation in forest soils probably due to the toxicity of NO_2_^−^ on CH_4_-oxidizing bacteria or increased NH_4_^+^ concentration following NO_3_^−^ addition^10^,^11^,^12^,^13^,^14^,^15^. The addition of glucose as a labile C source can increase the inhibition by NO_3_^−^-N of the soil CH_4_ oxidation^14^. The increase in atmospheric carbon dioxide concentration can promote the growth of vegetation, probably resulting in an increased C input into the soil via litter decomposition and turnover of roots. Furthermore, almost half of dissolved organic carbon (DOC) in water extracts of organic layers under temperate forest stands exists in the form of glucose-C (Table S1), which can be normally used by soil microbes. The labile C supply and the variations of soil moisture can stimulate soil microbial activity and the turnover of NO_3_^−^-N and NH_4_^+^-N in soil, and the increased carbon dioxide concentration can have different effects on atmospheric CH_4_ uptake in unsaturated upland soils^7^,^16^. The effect of N input on CH_4_ uptake appears to depend on the types of added N, but the effects of N addition in combination with C sources on the soil CH_4_ uptake are partly understood.

The relationship between CH_4_ uptake and DOC concentration in soil is generally elusive^17^. On one hand, the addition of labile C sources can stimulate heterotrophic microbial processes, which cause low oxygen concentration in soil and thus inhibits CH_4_ uptake. *Fender et al*.^14^ reported that the addition of glucose at a rate of 941.9 g C m^−2^ reduced CH_4_ uptake by 83% and, more intriguingly, stimulated the inhibiting effect of KNO_3_ fertilization on CH_4_ uptake in a temperate forest soil. On the other hand, a rapid decomposition of added glucose in the soil probably increases microbial N immobilization by consuming NO_3_^−^-N and NH_4_^+^, which can affect the soil CH_4_ uptake. *Xu & Inubushi*^13^ reported that the 15-day oxic pre-incubation following addition of glucose at a rate of 10 mg C g^−1^ dry soil, stimulated CH_4_ uptake rates in temperate volcanic forest soils and this stimulation was larger than 1-day oxic pre-incubation. Furthermore, the stimulating effect of glucose addition on the soil CH_4_ uptake varied with the types of forest vegetation, with the largest effect in the Pinus forest soil. Probably, this varying effect of glucose on CH_4_ uptake partly depends on the status of inorganic N in the soil following addition of glucose. However, to our knowledge, the roles of NH_4_^+^ and NO_3_^−^ in inhibiting CH_4_ uptake in forest soils in the presence of soil labile C pool are not known.

Future climatic change is likely to alter the frequency and intensity of soil drying-wetting and freezing-thawing events^1^,^18^. Wetting of dry soil and freezing-thawing processes can release labile C and N into the soil^19^,^20^, but their impacts on the soil CH_4_ flux are unknown because they involve methanogenesis and methanotrophy^21^,^22^. Wetting dry soil can stimulate atmospheric CH_4_ oxidation in unsaturated upland soils mainly by alleviating osmotic stress on soil methanotrophs^23^,^24^, but the effect of the availability of soil C and N upon wetting on soil CH_4_ oxidation is not known. *Wu et al*.^25^ reported that there was a significant increase in the CH_4_ uptake activity following thawing and this increase generally decreased by increasing soil moisture from 32 to 55% WFPS. Methane-oxidizing bacteria are actually facultative and can utilize organic C sources, such as DOC released by wetting and freezing, other than CH_4_^17^,^26^. *Zhu et al*.^27^ reported that the change in soil carbon availability during thawing period could affect the dynamic of CH_4_ flux from Antarctica soils under laboratory conditions. Furthermore, the release of labile N pools (e.g. NH_4_^+^-N) into the soil at thaw^20^ can partly limit the capacity of CH_4_ oxidation. Due to the many variables involved, understanding the mechanisms involving soil CH_4_ flux during wetting and thawing periods is problematic^22^. Thus, it is urgent to study the synergistic effect of N and C addition on the soil CH_4_ uptake during wetting and thawing periods.

Broadleaf and Korean pine mixed forest (BKPF) is the major component of forest ecosystems in Changbai Mountains, northeastern China. In such district, the mature mixed forest lies in climax community of forest succession, with a greater soil organic matter content and lower bulk density than an adjacent secondary white birch forest (WBF)^20^. Due to relatively lower vegetation coverage and phototaxis property, soil available nutrients, microbial properties and hydrothermal conditions under the white birch forest stand are different from those under the mature mixed forest. The water extracts of organic layer samples collected from the WBF stand contained relatively higher DOC and microbial degradable C pools (e.g. glucose-C) than those from the BKPF stand (Table S1 and Figure S1). The differences in properties of organic layers and mineral soils under the two forest stands may influence the responses of soil CH_4_ uptake to the addition of glucose and nitrogen as NH_4_Cl or KNO_3_. Furthermore, whether the increase in DOC input from autumn freshly fallen leaves and in combination with increased N deposition can affect the CH_4_ uptake by forest soils during soil wetting and thawing periods has been unknown so far^22^. We hypothesized that freezing treatment and glucose, as a DOC source, can affect the roles of NH_4_^+^ and NO_3_^−^ in inhibiting soil CH_4_ uptake. For this purpose, a series of laboratory incubation experiments were done to study (1) the single and interactive effect of C and N addition on CH_4_ uptake in WBF and KBPF soils without and with freezing treatment; (2) the main driving mechanisms of CH_4_ uptake during soil wetting and thawing periods by considering the variations of soil properties such as labile C and N pools. The results improve our understanding of how DOC input from forest organic layers and in combination with N deposition can affect the soil CH_4_ uptake under forest stands during soil wetting and thawing periods.

## Results

### Changes in soil properties

The addition of glucose alone reduced NH_4_^+^-N and NO_3_^−^-N concentrations in the WBF and BKPF soils without and with freezing treatment (*P* < 0.0001) (Tables S2–S4). The decrease in NH_4_^+^-N concentration induced by glucose addition after freezing was higher compared to the unfrozen soils (*P* < 0.05) (Tables S2 and S3). The addition of glucose alone increased MBC (*P* < 0.0001) and MBC:MBN ratios (*P* < 0.001) of the two forest soils without and with freezing treatment (Tables S2–S4). NH_4_^+^-N concentration in the two forest soils treated with KNO_3_ alone significantly increased without and with freezing treatment, especially in the WBF soil (*P* < 0.05) (Tables S2–S4). However, this increase did not occur in the KNO_3_+glucose treatment (Tables S2 and S3). The addition of NH_4_Cl and KNO_3_ alone significantly decreased soil pH without and with freezing treatment (*P* < 0.0001), and this decrease varied with vegetation types (*P* < 0.01) (Tables S2–S4). Freezing treatment caused a release of DOC and DON (*P* < 0.0001) and a significant decrease in MBC and the MBC:MBN ratios of the two forest soils in all treatments (*P* < 0.001) (Tables S2–S4). The increased release of DOC induced by freezing varied with vegetation types (*P* < 0.001) (Table S4).

### Soil CH_4_ uptake without and with freezing

**Figure 1:**
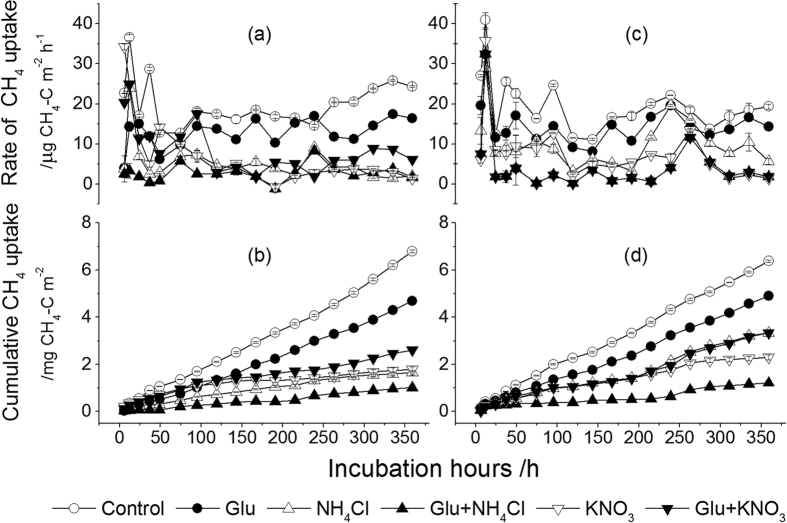
Effect of C and N addition on instantaneous rates of CH_4_ uptake and cumulative CH_4_ uptake in the WBF and BKPF soils without freezing treatment during the 15-day incubation. (**a,b**) present WBF soil; (**c,d**) present BKPF soil. Error bars represent standard error.

**Figure 2:**
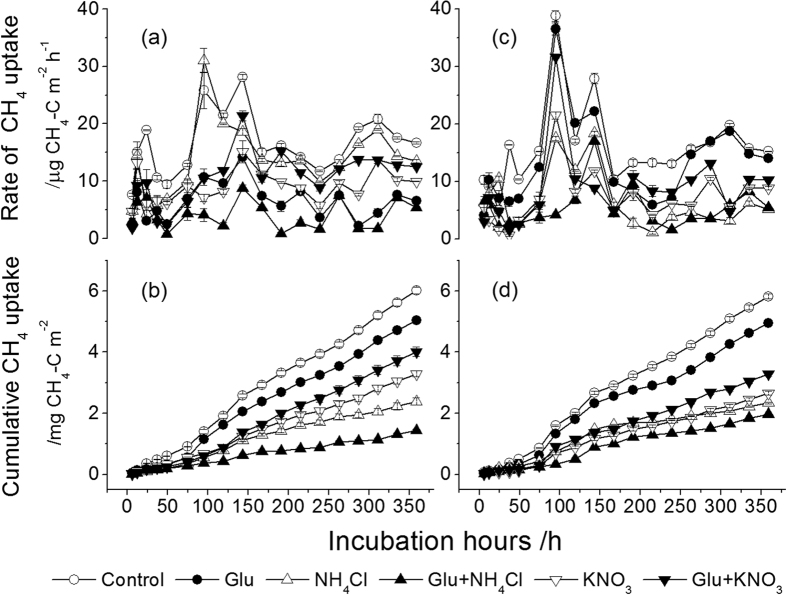
Effect of C and N addition on instantaneous rates of CH_4_ uptake and cumulative CH_4_ uptake in the WBF and BKPF soils with freezing treatment during the 15-day incubation. (**a,b**) present WBF soil; (**c,d**) present BKPF soil. Error bars represent standard error.

Without freezing treatment, an increased uptake of CH_4_ was observed immediately after wetting the WBF and BKPF soils in the control (Fig. 1a,c). The rate of CH_4_ uptake in the control was almost the highest among all treatments during the 15-day incubation, thus resulting in significantly higher cumulative CH_4_ uptake than that from soils treated with Glu, NH_4_Cl or KNO_3_ alone at the end of incubation (*P* < 0.0001) (Fig. 1b,d and Table 1). Compared to the unfrozen soils (Fig. 1a,c), the peak of CH_4_ uptake was delayed and occurred generally within 95 h to 143 h after the beginning of thaw (Fig. 2a,c). The cumulative CH_4_ uptake in the control after freezing was significantly higher than that from soils treated with Glu, NH_4_Cl or KNO_3_ alone during the 15-day incubation (*P* < 0.0001) (Fig. 2b,d and Table 1). Without and with freezing treatment, the cumulative CH_4_ uptake in the two forest soils treated with glucose alone and in combination with N sources could be ranked as Glu < Glu+KNO_3_ < Glu+NH_4_Cl (*P* < 0.05), except no difference between Glu and Glu+KNO_3_ in the WBF soil after freezing (Figs 1 and 2).

ANOVA analysis showed that the cumulative CH_4_ uptake without freezing was affected by the addition of NH_4_Cl or KNO_3_ and glucose singularly and interactively (*P* < 0.0001), and the interaction effect varied with forest types and incubation time (*P* < 0.001) (Table 1). However, only the addition of KNO_3_ and glucose interactively affected the cumulative CH_4_ uptake with freezing (*P* < 0.0001) (Table 1).

**Figure 3:**
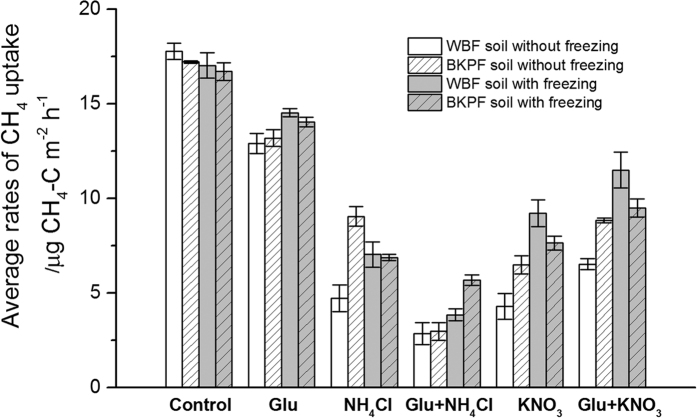
Effect of C and N addition on the average rates of CH_4_ uptake in the WBF and BKPF soils without and with freezing treatment during the 15-day incubation. Error bars represent standard error.

The average rates of CH_4_ uptake in the control without and with freezing were higher than those in the soils treated with C and N alone and in combination during the 15-day incubation, and the lowest rate occurred in the Glu+NH_4_Cl treatment (*P* < 0.05) (Fig. 3 and Table 2). The addition of NH_4_Cl, KNO_3_, and KNO_3_ plus glucose without freezing resulted in a smaller average rate of CH_4_ uptake in the WBF soil than in the BKPF soil during the 15-day incubation (*P* < 0.05) (Fig. 3). However, only the Glu+NH_4_Cl treatment had a lower rate of CH_4_ uptake in the WBF soil than that in the BKPF soil with freezing (*P* < 0.05) (Fig. 3). ANOVA analysis showed that the inhibition of NH_4_Cl or KNO_3_ on the average rates of CH_4_ uptake varied with vegetation types (*P* < 0.05) and freezing treatment (*P* < 0.05) (Table 2).

### The inhibition by N addition of CH_4_ uptake in forest soils

The effects of glucose addition and freezing on the inhibition by N addition of CH_4_ uptake in forest soils were shown in Table 3. In the absence of freezing and glucose supply, the relative inhibition of CH_4_ uptake induced by NH_4_Cl and KNO_3_ was both significantly greater in the WBF soil than that in the BKPF soil (*P* < 0.05). Without glucose, the freezing treatment significantly decreased the absolute and relative inhibition of N addition as NH_4_Cl and KNO_3_ on CH_4_ uptake in the WBF soil, while it significantly increased the absolute and relative inhibition by NH_4_Cl addition of the CH_4_ uptake in the BKPF soil (*P* < 0.05).

Glucose addition significantly increased the relative inhibition by NH_4_Cl of the CH_4_ uptake in the WBF soil with freezing and in the BKPF soil without freezing (*P* < 0.05) (Table 3). However, glucose addition significantly reduced the absolute and relative inhibition of KNO_3_ addition on CH_4_ uptake in the two forest soils with and without freezing (*P* < 0.0001) (Tables 3 and 4). ANOVA analysis showed that freezing treatment and in combination with glucose and vegetation type could significantly affect the inhibition of NH_4_Cl addition on the CH_4_ uptake (*P* < 0.05), and that the inhibition of KNO_3_ addition on the CH_4_ uptake was influenced by freezing and glucose supply (*P* < 0.0001) (Table 4).

### Relationships between CH_4_ uptake and soil properties

The average rates of CH_4_ uptake in forest soils without and with freezing treatment were both positively correlated with soil pH (*P* < 0.001) and negatively correlated with the concentrations of soil NH_4_^+^-N and DON (*P* < 0.001) (Table 5). According to the results of stepwise regression analysis, 68% of the variability in the soil CH_4_ uptake without freezing could be attributed to the soil pH and NH_4_^+^-N, with the predominant influence of soil pH (Table 6). Meanwhile, 59% of the variability in the soil CH_4_ uptake with freezing treatment could be explained by the soil NH_4_^+^N and NO_3_^−^-N, and affected by the NH_4_^+^-N mostly (Table 6). Together with soil samples without and with freezing, 66% of the variability in the soil CH_4_ uptake could be explained by the soil pH, inorganic N and DOC, and affected by the NH_4_^+^-N mostly (Table 6).

## Discussion

### Effect of freezing on CH_4_ uptake in forest soils

Thawing of frozen soils in the absence of C and N addition significantly increased the release of NH_4_^+^-N and DOC into the soils, compared to the unfrozen soils (Tables S2 and S3), and this may reduce CH_4_ uptake in forest soils during thawing period^5^,^26^. The release of labile C and N into the soil after thaw resulted in a significant decrease in cumulative CH_4_ uptake in the control throughout the experimental period (Figs 1 and 2). Previous laboratory studies involving freeze-thaw effects have seldomly compared with non-freezing experiment, and in field measurement, the effect of freeze-thaw cycle on soil CH_4_ uptake was quite variable. Many previous studies showed that freeze-thaw cycle significantly decreased soil CH_4_ uptake in a desert grassland^28^ and a northern hardwood forest^29^. However, *Borken et al*.^30^ observed that soil freezing by snow removal increased the rates of soil CH_4_ uptake in a temperate forest. And no changes in CH_4_ uptake due to freeze-thaw cycle also occurred in a grassland^31^. *Gao et al*.^32^ reported that serious freezing at −15 °C significantly decreased the cumulative CH_4_ uptake rate in a alpine meadow soil but mild freezing at −5 °C had a similar rate compared to the non-freezing treatment. Probably, freezing conditions and the resultant changes in soil gas diffusion and the release of labile C and N pools lead to different changes in CH_4_ uptake during thawing period.

Soil moisture is the primary control on the soil CH_4_ oxidation^33^, either by affecting gas diffusion or because low soil moisture can cause osmotic stress on soil methanotrophs^23^,^34^. Soil cores were incubated at 55% WFPS, and thus gas diffusion was not a possible limitation factor of the CH_4_ uptake because gas diffusion through soil is restricted over 65% WFPS^35^. Probably, soil CH_4_ uptake after freezing under the experimental conditions was attributed to the changes in soil inorganic N and DOC pools and the properties of methanotrophs. At the onset of thaw, the soil CH_4_ uptake across all the treatments was smaller but it increased significantly four to six days after thaw (Fig. 2a,c), and the pulse CH_4_ uptake after frost was similar to that within 12 h after wetting (Fig. 1a,c). This indicated that there was a delay of several days for a complete reactivation of inactive methanotrophs in the soils frozen at −18 °C for 50 days. This delay probably resulted from the initial pulse release of dissolved organic C (e.g. organic acids) shortly after thaw to be used by facultative methanotrophs^26^. This can limit the recovery of methanotrophs which are capable of oxidizing atmospheric CH_4_. Together with the release of DOC and inorganic N into the soil after severe freezing (Tables S2 and S3) and the changes in soil microbial community as indicated by the microbial biomass C-to-N ratio (Table S4), further research should characterize quality and quantity of DOC and labile N released into the soil at thaw and their relationships to the soil CH_4_ uptake as well as the functions of soil methanotrophs^22^.

### Effect of glucose addition alone on CH_4_ uptake in forest soils

Glucose is a labile C source that can be easily used by methanogenic bacteria to produce CH_4_ and by other microorganisms producing CO_2_ and consuming O_2_, probably resulting in the inhibition of CH_4_ uptake. Alternatively, the addition of glucose can increase N immobilization in forest soils and reduce NO_3_^−^-N concentration following preincubation, and this can stimulate CH_4_ oxidation^13^. Methanotrphs can utilize multi-C compounds (e.g. glucose) as sole sources of C and energy in the absence of methane^17^,^36^. The changes induced by the labile C can thus affect the soil CH_4_ uptake. Without preincubation, the addition of glucose alone at a rate of 6.4 g C m^−2^ significantly inhibited the cumulative atmospheric CH_4_ uptake by 23% to 31% in the two forest soils during wetting period (Fig. 1c,d), and this agrees with what reported by *Fender at al*.^14^. The reason of the inhibition effect of glucose may be the increased microbial respiration^37^ and microbial biomass C caused by the added glucose (Table S2) with the decrease in O_2_ concentration of soil thus limiting CH_4_ oxidation^14^. Additionally, the resultant anaerobic environment in soil micro-sites can stimulate denitrification process with consumption of NO_3_^−^; the intermediate product NO_2_^−^ of denitrification is toxic to CH_4_-oxidizing bacteria^10^,^38^. During thawing period, the inhibition of glucose on the cumulative soil CH_4_ uptake was reduced by approximately 15% (Fig. 2c,d), which was significantly smaller than that during wetting period (Fig. 1c,d). Probably, the more release of DOC into the soil after frost (Tables S2 and S3) reduced the inhibition of CH_4_ uptake in the two forest soils by glucose addition, particularly in the WBF soil (Fig. 3). This is probably related to the relatively high labile C pools (e.g. glucose-C and protein-like substance) in water extracts of forest organic layers under WBF stand than under BKPF stand (Table S1 and Figure S1). According to *Wieczorek et al*.^26^, the facultative methanotrophs can change their substrate utilization in the presence of different organic C sources and this may reduce CH_4_ oxidation. The addition of glucose alone caused a significant increase of MBC:MBN ratios in the forest soils with and without freezing (Tables S2–S4), indicating a shift in microbial community towards more fungal (average C:N ratio, 5–15) than bacteria (average C:N ratio, 3–6)^39^,^40^. This shift can support the inhibition of glucose on soil CH_4_ uptake, because a negative correlation between methane-oxidizing bacteria and fungal biomass was observed in forest soils^41^.

Contrary to the inhibition of CH_4_ uptake by glucose addition, *Xu & Inubushi*^13^ reported that adding glucose at a rate of 10 mg g^−1^ dry soil significantly stimulated CH_4_ uptake in the volcanic forest soils, especially in the *Pinus* forest soil, with a relatively low efficiency in utilizing organic C, and the stimulation depended on forest types and preincubation conditions. *Sullivan et al*.^17^ reported a positive correlation (*r* = 0.76, *P* < 0.01) between soil DOC concentration and CH_4_ oxidation rate and indicated DOC as an important regulator of CH_4_ oxidation in arid soils. Incubation experiment conducted by *Hilger et al*.^42^ showed that glucose concentration was positively correlated to CH_4_ uptake in a landfill cover soil (*r* = 0.94, *P* < 0.05). In our study, there was a significant negative correlation between the average CH_4_ uptake without freezing treatment and the soil DOC concentration but not after frost (Table 5). The results by *Burke et al*.^41^ indicated that methane-oxidizing bacteria are more likely found in areas with low C and nutrient cycling rates. According to the results of ANOVA analysis, the inhibition of glucose addition on the soil CH_4_ uptake was affected by forest vegetation, freezing, and types of added N (Tables 1 and 2). From the results of this study and previous studies, it can be thus reasonably concluded that the addition of external C such as glucose has variable impacts on CH_4_ uptake in unsaturated soils, which depends on microbial C utilization, soil N availability, and hydrothermal conditions. More interesting, the content of glucose in water extracts of organic layers under the WBF and BKPF stands ranged from 2.4 to 4.3 g C m^−2^, accounting for 40% to 55% of DOC pool, and forest organic layers under the WBF stand contained the relatively higher DOC and microbial degradable C pools (Table S4 and Figure S1). Due to the fact that the responses of CH_4_ uptake to the addition of glucose varied with forest vegetation and freezing treatment (Tables 1 and 2), the experimental results indicated that the quality and quantity of DOC released from forest organic layers into the soil can partly affect the soil CH_4_ uptake under the two forest stands, especially during spring thawing after winter sincere freezing.

### Effect of N sources and in combination with glucose on CH_4_ uptake in forest soils

There was a significant negative relationship between the average rates of CH_4_ uptake and NH_4_^+^-N concentration of forest soils in all treatments without and with freezing (Table 5), which showed the inhibition effect of NH_4_^+^-N on the CH_4_ uptake in unsaturated forest soils^22^,^43^. The competition of NH_4_^+^ with CH_4_ on the enzyme responsible for both oxidations is considered the reason for the inhibition effect of NH_4_^+^ on the soil CH_4_ uptake^4^,^5^,^12^; in addition the concomitant conversion of NH_4_^+^ to NH_2_OH and NO_2_^−^ would be toxic to CH_4_-oxidizing bacteria^10^,^38^. This hypothesis was confirmed by *Xu & Inubushi*^12^, who reported that the use of nitrification inhibitor eliminated the inhibition of CH_4_ uptake by NH_4_^+^. Hence, the turnover rather than concentration of NH_4_^+^ can influence the soil CH_4_ uptake under the experimental conditions^3^,^5^. The smallest average rates of CH_4_ uptake occurred in the NH_4_Cl+Glu treatment, with significant differences in the BKPF soil without and with freezing and in the WBF soil without freezing compared to the NH_4_Cl treatment and Glu addition alone (Fig. 3). The results indicated that there was a positive synergistic inhibition effect of NH_4_^+^-N and glucose on the soil CH_4_ uptake under the experimental conditions and this synergistic effect varied with forest types and freezing treatment.

Besides NH_4_^+^-N effect on the soil CH_4_ uptake, the significant decrease in soil pH upon N addition without and with freezing (Tables S2–S4) also decreased the soil CH_4_ uptake (Table 5)^12^,^44^,^45^. Negative effects of soil acidification on soil physical and chemical properties (e.g. Al^3+^) and microbial activities have the potential to reduce CH_4_ uptake in forest soils^44^,^45^.

Nitrate-N addition has a strong inhibition of CH_4_ uptake in unsaturated forest soil^8^,^10^,^11^,^12^,^13^,^15^, and this inhibition varied with vegetation types and freezing treatment (Table 2). An increase in NH_4_^+^-N concentration without and with freezing treatment occurred in the two forest soils treated with KNO_3_ alone, particularly in the WBF soil (Tables S2–S4), and there was a significant negative correlation between the average rate of CH_4_ uptake and the NH_4_^+^-N concentration in the soils not treated with NH_4_Cl (*r* = −0.533, *P* < 0.01). The inhibition of CH_4_ uptake by KNO_3_ under the experimental conditions can thus depend on the accumulation of NH_4_^+^ upon KNO_3_ addition, which was also proposed by *Fender et al*.^14^. However, *Wang & Ineson*^10^ did not show a significant change in NH_4_^+^-N concentration after KNO_3_ addition and indicated that NO_3_^−^-N rather than NH_4_^+^-N nor K^+^ was the major responsible inhibitory component for the soil CH_4_ uptake. Based on significant differences in concentrations of NH_4_^+^-N and MBN between the Glu treatment and Glu+KNO_3_ treatment after freezing (Table S3), it was assumed that NO_3_^−^-N was partly converted into NH_4_^+^-N in forest soil at thaw and that microbial N immobilization preferable for NH_4_^+^-N was increased by adding C. Due to the rapid immobilization of NO_3_^−^ in forest soils^46^, DON concentration of the two forest soils with and without freezing was significantly increased by adding NO_3_^−^-N, and the response varied with vegetation type and freezing treatment (Tables S2–S4). Probably, the conversion of NO_3_^−^ to NH_4_^+^ under the experimental conditions resulted from the mineralization of increased DON in the soils. Further research should characterize the accumulation of NH_4_^+^-N upon NO_3_^−^-N addition in forest soils with varying microbial C availability using label ^15^N technology and its relationship to the soil CH_4_ uptake.

Under the experimental conditions, the addition of glucose significantly weakened the inhibition effect of NO_3_^−^-N on the CH_4_ uptake in the two forest soils without and with freezing (Tables 3 and 4). Simultaneously, compared to the KNO_3_ treatment, NH_4_^+^-N concentration in the KNO_3_+Glu treatment significantly decreased in the two forest soils with freezing, particularly in the WBF soil (Table S3), and this N concentration was also decreased in the WBF soil without freezing (Table S2). It further supports that NH_4_^+^-N rather than NO_3_^−^-N itself in the presence of labile C has an inhibition effect on the soil CH_4_ uptake. However, *Fender et al*.^14^ demonstrated that glucose addition aggravated the inhibition effect of KNO_3_ on the CH_4_ uptake in forest soil from 86% to 99.4%. In spite of that, they found an increase in NH_4_^+^-N content compared to the soil treated with NO_3_^−^-N alone, which is still coincided with the suppose: indirect inhibition effect of NO_3_^−^-N on the soil CH_4_ uptake by transforming to NH_4_^+^-N. Besides the NH_4_^+^-N, the decline of soil pH upon NO_3_^−^ addition can be considered a cause of NO_3_^−^-N inhibition^44^. However, *Mochizuki et al*.^15^ reported that the decrease in soil pH accompanied by the addition of nitrate was not responsible for the strong inhibition by nitrate of CH_4_ oxidation. Recently, *in situ* atmospheric CH_4_ oxidation rates in temperate forests from South Korea were reported to be positively correlated with soil nitrate concentration, and the short-term experimental addition of NO_3_^−^-N significantly stimulated the atmospheric CH_4_ oxidation but inhibited oxidation under high CH_4_ concentration^8^. Hence, the mechanisms involving the inhibition by NO_3_^−^-N of CH_4_ uptake need to be further studied.

Toxicity of NO_3_^−^ and NO_2_^−^ produced via NO_3_^−^ reduction in anaerobic microsites to CH_4_-oxidizing bacteria has been reported to explain the inhibitory effect of NO_3_^−^ addition on the soil CH_4_ uptake^10^,^38^. However, it is unreal under experimental conditions because the relatively low soil moisture (55% WFPS) and headspace aeration within PVC cylinder at each gas sampling could ensure enough oxygen concentration available in the soil. Certainly, it may create anaerobic environment temporarily after the addition of glucose, because high microbial respiration stimulated by glucose^37^ consumes large amounts of oxygen. But, according to simultaneous measurements of nitrous oxide emission from the same experiments, the KNO_3_+Glu treatment had no more cumulative nitrous oxide emission from the two forest soils than the Glu treatment during wetting and thawing periods (data not shown), which indicated the presence of glucose cannot stimulate the denitrification of nitrate-N in the soils at 55% WFPS.

In this study, freezing significantly influenced the inhibition effect of NO_3_^−^-N and NH_4_^+^-N on the soil CH_4_ uptake (Table 2). This was linked to the increase in soil DOC concentration and a decrease in the microbial biomass C-to-N ratios caused by freezing (Tables S2–S4). The decrease in the ratios shows that freezing can cause a shift in microbial composition towards more bacteria with lower microbial C-to-N ratios than fungi^47^. Together with the changes in soil labile C and N pools and pH, it can be concluded that changes in CH_4_ uptake in unsaturated forest soils without and with freezing treatment depended on soil pH, labile C, turnover of N, and microbial community structure.

The quantity and quality of DOC in water extracts of forest organic layers varies with forest types (Table S1 and Figure S1), and the input of the labile C into the soil may affect atmospheric CH_4_ uptake under forest stands. Our current studies showed that the addition of forest leaf litters at a dose of 0.0125 g g^−1^ oven-dried soil resulted in an increase of glucose-C concentration in the soil from 36.3 to 66.6 μg glucose-C g^−1^ oven-dried soil during freezing-thawing periods (data not shown). Together with the changes in inorganic N and labile C pools released into the soil after freezing as well as glucose-C as one important form of DOC in water-extracts of forest organic layers (Table S1), the varying synergistic effect of inorganic N and glucose-C addition on soil CH_4_ uptake suggested that DOC input from forest organic layers can change the inhibition effect of N deposition on the soil atmospheric CH_4_ uptake, which depends on the types of deposited N.

## Methods

### Site description and collection of forest soil and organic layer samples

The studying area locates near the National Research Station of Changbai Mountain Forestry Ecosystem (42°24′ N, 128°6′ E) in Jilin Province, northeastern China with a typical continental temperature climate. The average elevation of the area is 738 m with a flat topography. Based on regular meteorological measurements of the station during the period from 1982 to 2012, daily average air temperature and surface soil temperature in the field from late November to next early March generally rang from −5 °C to −30 °C, and snow depth in winter is normally within the range from 5 cm to 35 cm. For this reason, soil profile in winter can be frozen down to 1.0–1.5 m depth and a complete disappearance of such frozen soil layer normally occurs in the middle of May each year^20^. A mature broadleaf and Korean pine mixed forest and an adjacent white birch forest were selected for soil sampling. The former is of multi-layer structure with canopy density of 0.8 and the average age of dominating trees is about 200 years old; the later as a secondary forest has a more simple structure with canopy density of 0.6 and the average age of dominating trees is about 70 years. Due to relatively lower vegetation coverage and phototaxis property, soil moisture under the white birch forest stand is smaller than that under the mature mixed forest over the year, and the former is characterized by the relatively higher frequency of freezing and thawing cycles during non-growth season period. To collect composite forest soil and organic layer samples, eighteen 1 m × 1 m plots were selected in each forest stand in October 2012. Mineral soil samples (0–10 cm) in each plot were collected using an 8-cm diameter auger after removing the ground surface mulch, and organic layer samples including fresh and semi-decomposed litter were collected. All samples were kept separately in air-tight plastic bags and rapidly transported to the laboratory within 24 h. The soil samples from each forest stand were mixed thoroughly, sieved (<2 mm) to remove small stones and debris, and then stored in the dark at 4 °C prior to incubation and analysis of soil properties. The organic layer samples from each forest stand were dried at 60 °C for 48 h and milled for measurements of plant sample properties.

### Measurements of properties of forest organic layers and mineral soils

Triplicate soils were dried at 105 °C for 24 h to determine moisture content. Fresh soil pH (soil/water, 1/2.5, *w*/*w*) and pH values in water extracts of forest organic layers (sample/water, 1/10, *w/w*) were respectively measured with a portable pH meter (PB-10, Sartorius, Germany). Total C and N concentrations in forest organic layers and soil samples were measured using an elemental analyzer (vario Macro cube, Elementar, Germany). Concentrations of soil microbial biomass C (MBC) and N (MBN) were measured by the chloroform fumigation and extraction method^48^,^49^. Fresh forest mineral soils (5.0 g) were extracted by shaking with 25 mL of 0.5 mol L^−1^ K_2_SO_4_ solution for 30 min and dried organic layer samples (5.0 g) by shaking with 50 mL of deionized water for 24 h on an end-over-end shaker. The suspensions were centrifuged at 4500 ***g*** for 5 min and then filtered into 50-mL plastic bottles via cellulose-acetate membrane filters (0.45 μm pore size). Concentrations of NH_4_^+^-N, NO_3_^−^-N, total N (TN), and DOC in the soil extracts and DOC in the organic layer extracts were measured using a continuous flow analyzer (SAN^++^, SKALAR, the Netherlands). Concentrations of soil dissolved organic N (DON) were calculated according to the differences between TN and mineral N (NH_4_^+^-N and NO_3_^−^-N) concentrations in soil extracts. The soil MBC and MBN were calculated by the differences of K_2_SO_4_-extractable DOC and TN pools between fumigated and non-fumigated soils and divided by 0.45^48^,^49^,^50^, assuming that fumigation causes a release of microbial N in the same proportion as for microbial C. The glucose concentrations in water extracts of forest organic layers were determined by anthrone-sulfuric acid colorimetric method^51^. UV absorbance at 254 nm (UV_254_) of the water extracts was measured using a spectrophotometer (UV-2800A, Unico, USA). Water extracts of forest organic layers were diluted 40 times for measuring excitation-emission matrix (EEM) fluorescence spectra using a fluorometer (Fluoromax-4, Horiba, USA). For the EEMs, instrumental bias corrections were conducted with S/R model and inner filter corrections^52^ were carried out using absorbance spectrum measured with a spectrophotometer (U-2000, Hitachi, Japan). Then, after subtracting the EEM of Milli-water, EEMs of the organic layer water extracts were calibrated to the water Raman signal^53^ and expressed in Raman units (RU, nm^−1^) (Figure S1). Humification index (HIX) was calculated by the ratio of two integrated regions of emission scan (sum of 436 to 480 nm divided by the sum of 300 to 344 nm) with excitation at 255 nm, indicating the relative humification of forest organic layer extracts^54^. The three components which the three fluorescence peaks represent are cited from *Chen et al*.^55^. Properties of water extracts of forest organic layers sampled under WBF and BKPF stands were shown in Table S1.

### Setup of incubation experiments

Wetting (non-freezing) and freezing-thawing experiments were conducted during November 2012 to January 2013. Packed soil cores were made according to bulk densities of BKPF and WBF soils in the field (Table 7). A factorial design with two forest types (BKPF and WBF) and the addition of nutrients (glucose, namely Glu 6.4 g C m^−2^, NH_4_Cl, 4.5 g N m^−2^, KNO_3_, 4.5 g N m^−2^, Glu+NH_4_Cl, Glu+KNO_3_) was established for the two incubation experiments; no nutrient addition was considered as control. The amounts of added N and C were approximately fourfold annual wet N input and twice glucose concentration in water extracts of organic layers (Table S1) under the two study forests, respectively. Experiments were replicated three times, giving a total of 72 packed soil cores.

Homogenized fresh soils (85 g) were transferred into 100-mL stainless steel cylinders (diameter in 50.5 mm) as a soil core. For each core, appropriate nutrients were precisely sprayed with deionized water as solutions onto the homogenized soil before packing to reach a water-filled pore space (WFPS) level of 55%; this operation were accomplished within 1 hour. In the freezing-thawing experiment 36 soil cores were frozen at −18 °C for 50 days, and the remaining 36 cores were sealed in gastight PVC cylinders (760 mL) with a gas sampling port equipped with 3-way stopcock separately to initiate the non-freezing experiment. Three PVC cylinders without soil served as blank. A long duration of freezing at −18 °C was simulated according to a severe winter frost from late December to next February near the study area^20^. In accordance with air temperature of the study area in late spring and autumn when soil freezing-thawing cycles intensively occur in the field, the soil cores were incubated at 10 °C in two incubators (LRH250, Yiheng Instruments, China) for 15 days. Soil moisture at 55% WFPS was simulated according to field moisture at thaw under the two study forests. Deionized water was duly added for each soil core by weighting during the 15-day incubation to avoid evaporation. Gas sampling was performed from each soil core and the blank at 6, 12, 24, 37, 49, 75, 95, 119, 143, 167, 191, 215, 239, 263, 287, 311, 335, and 359 h after the incubation initiated, according to preliminary studies showing a linear decrease in headspace CH_4_ concentration within 24 h after closure. Headspace gas samples of 30 ml were collected using 50-ml polypropylene syringes equipped with 3-way stopcock. Each time when gas sampling finished, all PVC cylinders were immediately taken outdoor to be well ventilated for 20 min and then sealed to continue the incubation till the next sampling time. The concentrations of CH_4_ in headspace gas samples were quantified by a gas chromatograph (Agilent 7890A, Franklin, USA) equipped with a flame ionization detector. The detector responses were calibrated using a certified gas standard, which contains 2.11 μL L^−1^ CH_4_ in air. Main properties of soil cores including moisture, bulk density, pH, NH_4_^+^-N, NO_3_^−^-N, DON, DOC, MBC, and MBN were measured immediately when the last gas sampling finished (359 h), as mentioned above. Soil WFPS inside each core was calculated by soil bulk density and moisture^56^. After freezing at −18 °C for 50 days, all the soil cores from freezing experiment were placed separately inside PVC cylinders and immediately incubated at 10 °C to simulate the soil thawing process. Headspace gases sampling and measurements of headspace CH_4_ concentration and soil properties were conducted at the same times of the non-frozen experiment.

### Calculation and statistical analysis

Instantaneous rates of soil CH_4_ uptake were calculated from the differences of headspace CH_4_ concentration between the blank and each treatment divided by the period of time from sealing to gas sampling, and were expressed in μg CH_4_-C m^−2^ h^−1^. The cumulative uptakes of CH_4_ during the 15-day incubation were calculated as the sum of CH_4_ uptake for each sampling and were expressed in mg CH_4_-C m^−2^. The average rates of CH_4_ uptake during the 15-day incubation were calculated by the slopes of linear regressions of cumulative CH_4_ uptakes against the incubation time (determination coefficient of regression, *R*^2^ > 0.95), and were expressed in μg CH_4_-C m^−2^ h^−1^. Means and standard errors for three replicates were calculated. Partial distributed data were normalized prior to statistical analysis. The absolute inhibition by N addition of CH_4_ uptake was calculated by the differences of average rates of CH_4_ uptake in the presence and absence of N sources (NH_4_Cl or KNO_3_), and its relative inhibition was calculated by the absolute inhibition divided by the average rate of CH_4_ uptake in the absence of N addition.

All measured variables were examined for normality (Shapiro-Wilk test) and homogeneity (Levene’s test) of variance and transformed where necessary. We used four-factor repeated analysis of variance (ANOVA) with vegetation type, N (NH_4_Cl or KNO_3_) and Glu addition, and freezing as fixed factors to assess their influences on the average rate of CH_4_ uptake and soil properties. The another four-factor repeated ANOVA was used with vegetation type, N (NH_4_Cl or KNO_3_) and Glu addition as independent variables between subjects and with sampling time as independent variable within subjects, to assess their influences on the instant rate of CH_4_ uptake and cumulative CH_4_ uptake during the 15-day incubation. The three-factor repeated ANOVA with vegetation type, freezing and Glu addition as fixed factors was used to assess their impacts on the inhibition by NH_4_Cl and KNO_3_ of the soil CH_4_ uptake. Pearson correlation between soil properties and the average rate of CH_4_ uptake in forest soils without and with freezing was performed. Stepwise regression analysis was performed to assess the main soil properties which can affect the average rates of CH_4_ uptake without and with freezing. Significant effects between treatments in soil properties and CH_4_ uptake were determined at the *P* < 0.05 level using student *T*-test. All statistical analyses were conducted with the software SPSS for Windows (version 19.0, IBM Corp., USA).

## Additional Information

**How to cite this article**: Wu, H.H. *et al*. Synergistic effects of dissolved organic carbon and inorganic nitrogen on methane uptake in forest soils without and with freezing treatment. *Sci. Rep*. **6**, 32555; doi: 10.1038/srep32555 (2016).

## Supplementary Material

Supplementary Information

## Figures and Tables

**Table 1:** Summary of ANOVA with repeated measures for the instant rates of CH_4_ uptake and cumulative CH_4_ uptake in forest soils without and with freezing treatment.

Source of variation	Without freezing				With freezing treatment			
	Instant rate of CH_4_ uptake		Cumulative CH_4_ uptake		Instant rate of CH_4_ uptake		Cumulative CH_4_ uptake	
	*F*	*P*	*F*	*P*	*F*	*P*	*F*	*P*
*N addition as NH*_*4*_*Cl*								
Between subjects								
Vegetation (VT)	25.6057	**<0.0001**	131.9421	**<0.0001**	1.1760	0.2791	22.6105	**<0.0001**
N addition (N)	854.5645	**<0.0001**	2593.9278	**<0.0001**	1135.8867	**<0.0001**	3259.7753	**<0.0001**
Glucose (Glu)	203.3456	**<0.0001**	794.7950	**<0.0001**	85.5486	**<0.0001**	407.3302	**<0.0001**
VT × N	11.9317	**0.0006**	37.7731	**<0.0001**	9.1389	**0.0027**	51.7877	**<0.0001**
VT × Glu	0.3894	0.5331	1.7737	0.1840	13.1318	**0.0003**	0.8708	0.3515
N × Glu	0.0014	0.9700	16.5619	**0.0001**	0.0266	0.8705	1.4048	0.2369
VT × N × Glu	14.1612	**0.0002**	13.4113	**0.0003**	4.7737	**0.0297**	0.9401	0.3331
Within subjects								
Time	19.5037	**<0.0001**	409.9718	**<0.0001**	42.9859	**<0.0001**	980.3995	**<0.0001**
VT × Time	6.0406	**<0.0001**	2.1556	**0.0055**	4.0185	**<0.0001**	1.4840	0.0992
N × Time	3.8538	**<0.0001**	16.8531	**<0.0001**	12.3712	**<0.0001**	53.7808	**<0.0001**
Glu × Time	1.7539	**0.0336**	2.3868	**0.0018**	1.6525	0.0512	5.0300	**<0.0001**
VT × N × Time	2.3838	**0.0019**	0.9377	0.5298	3.6115	**<0.0001**	1.8241	**0.0249**
VT × Glu × Time	3.6389	**<0.0001**	2.1149	**0.0067**	2.1210	**0.0065**	1.1560	0.3006
N × Glu × Time	2.0626	**0.0086**	2.5856	**0.0007**	4.1774	**<0.0001**	3.5101	**<0.0001**
VT × N × Glu × Time	1.3216	0.1775	0.9395	0.5278	5.7621	**<0.0001**	1.7457	**0.0348**
*N addition as KNO*_*3*_								
Between subjects								
Vegetation (VT)	9.5220	**0.0022**	13.3708	**0.0003**	42.3268	**<0.0001**	50.1128	**<0.0001**
N addition (N)	925.2851	**<0.0001**	2078.8460	**<0.0001**	593.6729	**<0.0001**	1643.8224	**<0.0001**
Glucose (Glu)	19.0963	**<0.0001**	193.7710	**<0.0001**	7.9758	**0.0051**	43.4155	**<0.0001**
VT × N	1.0196	0.3135	20.6737	**<0.0001**	14.8393	**0.0001**	22.7903	**<0.0001**
VT × Glu	4.9481	**0.0269**	7.8644	**0.0054**	1.1113	0.2927	9.8260	**0.0019**
N × Glu	207.7277	**<0.0001**	502.5143	**<0.0001**	70.4478	**<0.0001**	195.2618	**<0.0001**
VT × N × Glu	3.5933	0.0590	16.4131	**0.0001**	0.0179	0.8937	2.4216	0.1208
Within subjects								
Time	46.5006	**<0.0001**	987.0992	**<0.0001**	71.7421	**<0.0001**	913.3450	**<0.0001**
VT × Time	9.9547	**<0.0001**	2.8894	**0.0001**	11.4625	**<0.0001**	3.0983	**<0.0001**
N × Time	15.3293	**<0.0001**	52.4922	**<0.0001**	5.1846	**<0.0001**	24.4530	**<0.0001**
Glu × Time	6.0925	**<0.0001**	1.4020	0.1340	4.1249	**<0.0001**	1.4420	0.1159
VT × N × Time	15.0997	**<0.0001**	9.8376	**<0.0001**	5.0622	**<0.0001**	0.6866	0.8158
VT × Glu × Time	3.2209	**<0.0001**	1.1845	0.2761	2.3494	**0.0022**	0.7016	0.8009
N × Glu × Time	4.8770	**<0.0001**	7.6688	**<0.0001**	2.6330	**0.0005**	6.4203	**<0.0001**
VT × N × Glu × Time	1.7445	**0.0350**	0.2953	0.9975	2.7847	**0.0002**	0.7562	0.7427

**Table 2:** Summary of ANOVA with repeated measures for the average rates of CH_4_ uptake in forest soils during the 15-day incubation without and with freezing treatment.

Source of variation	N addition as NH_4_Cl		N addition as KNO_3_	
	*F*	*P*	*F*	*P*
Vegetation (VT)	0.1635	0.6887	0.0310	0.8614
N addition (N)	308.3975	**<0.0001**	351.7982	**<0.0001**
Glucose (Glu)	93.8161	**<0.0001**	5.4137	**0.0265**
Freezing (F)	4.9874	**0.0327**	22.6748	**0.0000**
VT × N	8.8145	**0.0056**	6.7330	**0.0142**
VT × Glu	0.7289	0.3996	1.3515	0.2536
VT × F	0.1035	0.7498	9.6477	**0.0040**
N × Glu	0.3905	0.5365	81.3734	**<0.0001**
N × F	4.2068	**0.0485**	18.9566	**0.0001**
Glu × F	10.6002	**0.0027**	0.7855	0.3821
VT × N × Glu	1.5187	0.2268	1.1876	0.2840
VT × N × F	0.1430	0.7078	10.0363	**0.0034**
VT × Glu × F	0.7038	0.4077	0.3218	0.5745
N × Glu × F	0.0151	0.9030	7.3364	**0.0108**
VT × N × Glu × F	5.5761	**0.0245**	1.1581	0.2899

**Table 3:** Effect of freezing treatment and glucose addition on the inhibition of CH_4_ uptake by N sources in forest soils.

Freezing treatment	Glucose addition	Average rates of CH_4_ uptake in the WBF soil				Average rates of CH_4_ uptake in the BKPF soil			
		NH_4_Cl-induced		KNO_3_-induced		NH_4_Cl-induced		KNO_3_-induced	
		Absolute inhibition /μg CH_4_-C m^−2^ h^−1^	Relative inhibition /%	Absolute inhibition /μg CH_4_-C m^−2^ h^−1^	Relative inhibition/%	Absolute inhibition/μg CH_4_-C m^−2^ h^−1^	Relative inhibition/%	Absolute inhibition/μg CH_4_-C m^−2^ h^−1^	Relative inhibition/%
Without freezing	No glucose	12.31 ± 0.71	72.33 ± 4.17	12.74 ± 0.68	74.86 ± 3.97	7.98 ± 0.51	46.85 ± 2.99	10.54 ± 0.48	61.94 ± 2.83
	With glucose	11.68 ± 0.57	80.42 ± 3.94	8.01 ± 0.30	55.12 ± 2.04	11.57 ± 0.47	79.61 ± 3.22	5.69 ± 0.12	39.14 ± 0.85
With freezing	No glucose	9.99 ± 0.66	58.70 ± 3.89	7.82 ± 0.71	45.91 ± 4.17	10.15 ± 0.17	59.65 ± 1.00	9.39 ± 0.36	55.14 ± 2.14
	With glucose	10.69 ± 0.32	73.55 ± 2.22	3.03 ± 0.96	20.88 ± 6.58	8.85 ± 0.29	60.94 ± 1.97	5.04 ± 0.48	34.69 ± 3.30

**Table 4:** Summary of ANOVA with repeated measures for the inhibition by N sources on the average rates of CH_4_ uptake in forest soils without and with freezing treatment.

Source of variation	Absolute inhibition of CH_4_ uptake		Relative inhibition of CH_4_ uptake	
	*F*	*P*	*F*	*P*
Inhibition by NH_4_Cl				
Vegetation (VT)	19.1043	**0.0005**	19.8412	**0.0004**
Freezing (F)	7.5539	**0.0143**	9.7710	**0.0065**
Glucose (Glu)	2.8238	0.1123	45.1664	**0.0000**
VT × F	3.9472	0.0643	3.2305	0.0912
VT × Glu	2.5206	0.1319	1.7649	0.2027
F × Glu	6.4774	**0.0216**	8.8183	**0.0090**
VT × F × Glu	19.6606	**0.0004**	19.8412	**0.0004**
Inhibition by KNO_3_				
Vegetation (VT)	0.3459	0.5647	0.3836	0.5444
Freezing (F)	53.2792	**<0.0001**	52.8470	**<0.0001**
Glucose (Glu)	136.2188	**<0.0001**	73.5058	**<0.0001**
VT × F	25.4935	**0.0001**	26.1945	**0.0001**
VT × Glu	0.0385	0.8469	0.0266	0.8726
F × Glu	0.0831	0.7768	0.1286	0.7246
VT × F × Glu	0.1217	0.7318	0.6642	0.4271

**Table 5:** Pearson correlation (*r*) coefficients between soil properties and the average rates of CH_4_ uptake in forest soils without and with freezing treatment.

		WPFS	pH	NO_3_^−^-N	NH_4_^+^-N	DON	DOC	MBN	MBC	MBC:MBN ratio
CH_4_ uptake without freezing	*r*	−0.165	**0.801**	−0.306	−**0.559**	−**0.662**	−**0.401**	−0.070	−0.125	−0.098
	*P* value	0.337	<0.001	0.069	<0.001	<0.001	0.015	0.678	0.466	0.532
CH_4_ uptake with freezing	*r*	0.013	**0.603**	−0.119	−**0.679**	**−0.606**	0.043	0.102	0.025	**0.359**
	*P* value	0.940	<0.001	0.491	<0.001	<0.001	0.802	0.516	0.882	0.036

*P* value, the significant levels of Pearson correlation coefficients.

**Table 6:** Summary of the stepwise regression analysis for the relationships between the average rates of CH_4_ uptake against soil properties without and with freezing treatment.

Explanatory variable	Coefficient	Standard error	Relative contribution	*R*^2^ value	*F*	*P* value	Standard errors of estimates
	Average rates of CH_4_ uptake in the soils without freezing (*n* = 36) /μg CH_4_-C m^−2^ h^−1^						
Constant	−77.367	14.353		0.68	35.324	<0.0001	2.9964
pH	15.811	2.556	75.22%				
NH_4_^+^-N /g N m^−2^	−0.723	0.354	24.78%				
	Average rates of CH_4_ uptake in the soils with freezing (*n* = 36) /μg CH_4_-C m^−2^ h^−1^						
Constant	16.486	1.110		0.59	23.326	<0.0001	2.8817
NH_4_^+^-N /g N m^−2^	−2.596	0.385	67.08%				
NO_3_^−^-N /g N m^−2^	−1.097	0.331	32.92%				
Average rates of CH_4_ uptake in the soils with and without freezing (*n* = 72) /μg CH_4_-C m^−2^ h^−1^							
Constant	−40.639	14.777		0.66	32.036	<0.0001	2.8876
pH	9.169	2.460	29.88%				
NH_4_^+^-N /g N m^−2^	−1.624	0.341	34.98%				
NO_3_^−^-N /g N m^−2^	−0.671	0.305	15.98%				
DOC /g C m^−2^	0.604	0.175	19.16%				

*R*^2^ value, determination coefficient of regression.

**Table 7:** Main soil properties under the two study temperate forests.

Vegetation type	Moisture (%, w/w)	pH (water)	Total C (mg C g^−1^)	Total N (mg N g^−1^)	Bulk density (g cm^−3^)	NO_3_^−^-N(μg N g^−1^)	NH_4_^+^-N(μg N g^−1^)	DON(μg N g^−1^)	DOC(μg C g^−1^)	MBN(mg N kg^−1^)	MBC(mg C kg^−1^)	MBC:MBN ratio
WBF	31.7	5.67	9.43	0.75	0.73	17.7	20.7	26.9	176.6	312	1985	6.3
BKPF	50.7	5.87	11.78	0.92	0.64	39.4	4.8	28.2	167.7	226	1716	7.7
