# Corrigendum: Synergistic effects of dissolved organic carbon and inorganic nitrogen on methane uptake in forest soils without and with freezing treatment

**DOI:** 10.1038/srep46120

**Published:** 2017-04-05

**Authors:** Haohao Wu, Xingkai Xu, Cuntao Duan, Tuansheng Li, Weiguo Cheng

Scientific Reports 6: Article number: 3255510.1038/srep32555; published online: 08 30 2016; updated: 04 05 2017

This Article contains errors in Table 7, where incorrect values for ‘Total C’ and ‘Total N’ are given. The correct Table 7 appears below as Table 1.

## Figures and Tables

**Table 1:** Main soil properties under the two study temperate forests.

Vegetation type	Moisture (%, w/w)	pH (water)	Total C (mg C g^−1^)	Total N (mg N g^−1^)	Bulk density (g cm^−3^)	NO_3_^−^ N (μg N g^−1^)	NH_4_^+^-N (μg N g^−1^)	DON (μg N g^−1^)	DOC (μg C g^−1^)	MBN (mg N kg^−1^)	MBC (mg C kg^−1^)	MBC:MBN ratio
WBF	31.7	5.67	56.8	4.5	0.73	17.7	20.7	26.9	176.6	312	1985	6.3
BKPF	50.7	5.87	82.6	7.1	0.64	39.4	4.8	28.2	167.7	226	1716	7.7

