# Supplementary material for: Synergistic effects of dissolved organic carbon and inorganic nitrogen on methane uptake in forest soils without and with freezing treatment
Source: Sci Rep. 2016 Aug 30;6:32555. doi: 10.1038/srep32555 (PMC5004170; doi:10.1038/srep32555)
Supplement: Supplementary Information [file srep32555-s1.pdf]

ELECTRONIC SUPPLEMENTARY MATERIAL

Synergistic effects of dissolved organic carbon and inorganic nitrogen on methane uptake in forest soils without and with freezing treatment

HaoHao Wu [1,2], XingKai Xu [1*], CunTao Duan [1], TuanSheng Li[3] & WeiGuo Cheng [4]

[1] State Key Laboratory of Atmospheric Boundary Layer Physics and Atmospheric Chemistry, Institute of Atmospheric Physics, Chinese Academy of Sciences, Beijing 100029, China;

[2] Department of Atmospheric Chemistry and Environmental Science, College of Earth Science, University of Chinese Academy of Sciences, Beijing 100049, China;

[3] College of Earth Science and Resources, Chang'an University, Xi'an 710054, China;

[4] Faculty of Agriculture, Yamagata University, Tsuruoka 997-8555, Japan

[*] Corresponding author email: xingkai_xu@mail.iap.ac.cn; Phone: +86-10-82085510

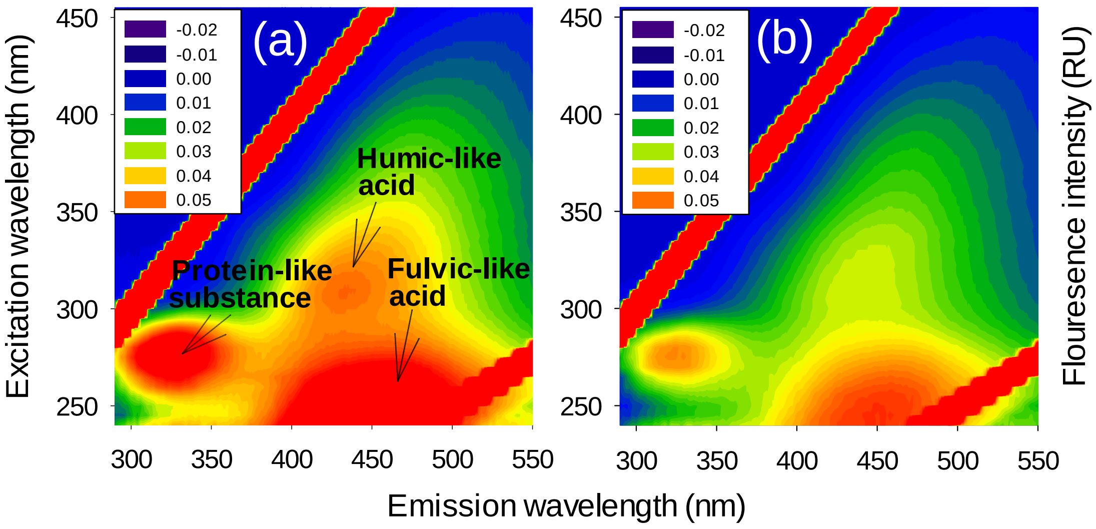

Figure S1 Excitation-emission matrix fluorescence spectra of water extracts (diluted 40 times) of organic layer samples collected under WBF (a) and BKPF (b) stands. The three components which the three fluorescence peaks represent are cited from *Chen et al.*[55].

Table S1 Properties of water extracts of organic layer samples collected under WBF and BKPF stands

	pH	DOC	Glucose	Glucose-C/DOC-C	UV_{254}	HIX
		g C m^{-2}	g C m^{-2}	%		
WBF	5.06	7.84±0.05	4.27±0.22	54.53±2.77	4.98	1.03
BKPF	4.99	5.65±0.05	2.41±0.28	39.59±4.60	5.00	2.49

Means of three replicates ± 1 x standard error.

Table S2 Effects of vegetation type and addition of C and N on labile C and N concentrations and pH values of soils after the 15-day incubation without freezing treatment

Vegetation type	C and N addition	pH (water)	K_2SO_4-extractable C and N pools /g m^{-2}				Microbial biomass /g m^{-2}		MBC:MBN ratios
			NO_3^--N	NH_4^+-N	DON	DOC	MBN	MBC	
WBF	Control	5.67±0.01	1.22±0.02	0.45±0.01	0.27±0.04	3.88±0.21	12.93±0.50	72.15±1.69	5.6±0.1
	Glucose (Glu)	5.76±0.01	0.95±0.04	0.12±0.00	0.09±0.02	3.79±0.14	12.95±0.45	86.79±3.73	6.7±0.1
	NH_4Cl	5.34±0.04	1.28±0.08	5.10±0.40	0.40±0.29	5.01±0.11	12.68±0.60	71.81±1.91	5.7±0.1
	Glu+NH_4Cl	5.34±0.02	1.25±0.09	3.35±0.11	1.05±0.01	5.02±0.15	12.50±0.51	79.20±1.89	6.4±0.1
	KNO_3	5.38±0.02	3.59±0.22	0.84±0.03	1.47±0.06	4.43±0.05	12.73±0.43	63.82±1.61	5.0±0.1
	Glu+KNO_3	5.43±0.01	3.95±0.19	0.37±0.02	1.33±0.20	4.31±0.08	13.43±0.46	87.79±2.69	6.5±0.0
BKPF	Control	5.87±0.02	1.30±0.02	0.35±0.02	0.07±0.01	4.21±0.04	7.35±0.06	41.59±0.21	5.7±0.0
	Glucose (Glu)	5.95±0.03	0.74±0.01	0.30±0.02	0.09±0.02	4.72±0.22	8.41±0.09	59.33±1.39	7.1±0.2
	NH_4Cl	5.36±0.01	1.40±0.06	2.54±0.40	0.91±0.15	4.26±0.19	9.30±1.17	43.96±0.82	5.4±0.6
	Glu+NH_4Cl	5.37±0.01	1.28±0.03	2.35±0.11	0.93±0.02	4.62±0.15	9.53±0.36	60.34±2.00	6.4±0.1
	KNO_3	5.35±0.01	5.42±0.27	0.37±0.02	0.20±0.04	4.30±0.20	7.45±0.62	39.01±0.14	5.5±0.5
	Glu+KNO_3	5.41±0.01	5.03±0.13	0.38±0.03	0.10±0.02	4.93±0.11	6.30±0.62	49.52±1.26	8.5±0.8

Means of three replicates ± 1 x standard error.

Table S3 Effects of vegetation type and addition of C and N on labile C and N concentrations and pH values of soils after the 15-day incubation with freezing treatment

Vegetation type	C and N addition	pH (water)	K_2SO_4-extractable C and N pools /g m^{-2}				Microbial biomass /g m^{-2}		MBC:MBN ratios
			NO_3^--N	NH_4^+-N	DON	DOC	MBN	MBC	
WBF	Control	5.44±0.01	0.77±0.01	1.25±0.00	1.03±0.06	9.74±0.11	11.34±0.05	57.98±0.62	5.1±0.0
	Glucose (Glu)	5.38±0.01	0.44±0.02	0.39±0.02	0.65±0.02	9.01±0.12	12.54±0.45	68.04±3.57	5.4±0.1
	NH_4Cl	5.31±0.02	0.57±0.02	3.51±0.33	1.19±0.05	9.62±0.26	12.89±0.31	50.55±1.64	3.9±0.0
	Glu+NH_4Cl	5.37±0.01	0.46±0.04	3.41±0.36	1.33±0.03	9.09±0.11	13.31±0.88	63.19±2.39	4.8±0.1
	KNO_3	5.33±0.01	4.13±0.10	1.65±0.02	1.18±0.04	9.63±0.03	13.54±0.67	57.99±2.74	4.3±0.0
	Glu+KNO_3	5.33±0.02	3.73±0.04	0.85±0.03	1.09±0.06	9.62±0.10	15.41±0.18	85.17±0.71	5.5±0.0
BKPF	Control	5.81±0.02	1.25±0.01	0.64±0.05	0.48±0.06	7.30±0.05	9.59±0.26	48.20±0.88	5.0±0.1
	Glucose (Glu)	5.91±0.03	0.52±0.02	0.20±0.01	0.46±0.02	7.09±0.10	8.29±0.21	54.70±1.04	6.6±0.1
	NH_4Cl	5.29±0.01	0.92±0.01	3.71±0.18	1.20±0.05	7.16±0.03	10.07±0.60	38.76±3.55	3.8±0.3
	Glu+NH_4Cl	5.29±0.02	0.84±0.01	2.55±0.03	1.10±0.01	7.57±0.10	9.96±0.80	53.21±0.71	5.7±0.6
	KNO_3	5.18±0.01	4.35±0.06	0.80±0.02	0.84±0.03	7.61±0.07	9.35±0.64	28.07±2.49	3.0±0.2
	Glu+KNO_3	5.37±0.02	3.55±0.03	0.57±0.03	0.65±0.03	8.09±0.12	10.72±0.30	32.83±1.21	3.1±0.1

Means of three replicates ± 1 x standard error.

Table S4 Summary of ANOVA with repeated measures for soil pH, inorganic N, DON, DOC, MBN, MBC, and MBC:MBN ratios without and with freezing treatment

Source of variation	pH		NO_3^--N		NH_4^+-N		DON		DOC		MBN		MBC		MBC:MBN ratio	
	F	*P*	*F*	*P*	*F*	*P*	*F*	*P*	*F*	*P*	*F*	*P*	*F*	*P*	*F*	*P*
N addition as NH_4Cl																
Vegetation (VT)	11.7370	**0.0017**	9.2958	**0.0046**	7.6259	**0.0094**	8.4298	**0.0066**	10.4086	**0.0029**	34.6544	**<0.0001**	90.5403	**<0.0001**	1.0429	0.3148
N addition (N)	104.8468	**<0.0001**	9.5387	**0.0041**	215.3531	**<0.0001**	84.6008	**<0.0001**	4.7169	**0.0374**	1.4605	0.2357	2.6921	0.1106	4.7674	**0.0364**
Glucose (Glu)	3.5825	0.0675	30.3250	**<0.0001**	33.9219	**<0.0001**	3.1830	0.0839	0.1544	0.6970	0.2197	0.6425	41.8389	**<0.0001**	18.4443	**0.0002**
Freezing (F)	7.4884	**0.0100**	73.6692	**<0.0001**	4.6094	**0.0395**	25.6904	**<0.0001**	156.3998	**<0.0001**	0.2229	0.6400	16.3593	**0.0003**	16.6519	**0.0003**
VT × N	10.4564	**0.0028**	1.3906	0.2470	3.7933	0.0603	0.0008	0.9772	4.5536	**0.0406**	0.1935	0.6629	0.6751	0.4174	0.2094	0.6503
VT × Glu	0.0040	0.9499	1.9203	0.1754	1.0325	0.3172	0.7884	0.3812	2.8386	0.1018	0.0242	0.8773	4.9673	**0.0330**	1.8445	0.1839
VT × F	0.8379	0.3668	10.7902	**0.0025**	0.0185	0.8925	0.1110	0.7412	16.9450	**0.0003**	1.1843	0.2846	15.2212	**0.0005**	1.0325	0.3172
N × Glu	0.3764	0.5439	9.9769	**0.0035**	2.7796	0.1052	0.2690	0.6076	0.5102	0.4802	0.1050	0.7480	0.0339	0.8552	0.0254	0.8744
N × F	0.0111	0.9168	10.0955	**0.0033**	5.1651	**0.0299**	4.1431	0.0502	3.3213	0.0777	0.4256	0.5188	2.1213	0.1550	1.7278	0.1980
Glu × F	0.0003	0.9852	0.6340	0.4318	0.2397	0.6278	0.8330	0.3682	1.5134	0.2276	0.4161	0.5235	1.5033	0.2291	0.0007	0.9784
VT × N × Glu	2.8301	0.1022	2.1170	0.1554	0.7116	0.4052	2.2753	0.1413	0.0311	0.8611	0.0149	0.9035	0.1459	0.7050	0.0703	0.7926
VT × N × F	3.5360	0.0692	0.1212	0.7300	12.9860	**0.0011**	0.3132	0.5796	5.7007	**0.0230**	0.4452	0.5094	0.5095	0.4805	0.0129	0.9102
VT × Glu × F	0.0887	0.7677	0.2402	0.6274	14.4350	**0.0006**	1.0950	0.3032	0.0850	0.7725	1.5350	0.2244	2.1659	0.1509	0.9498	0.3371
N × Glu × F	0.7649	0.3883	0.4186	0.5222	0.3591	0.5532	1.1994	0.2816	0.1996	0.6581	0.0901	0.7660	1.4901	0.2311	0.3689	0.5479
VT × N × Glu × F	0.9774	0.3303	0.0052	0.9430	0.2047	0.6540	0.7652	0.3882	0.0747	0.7864	0.1231	0.7280	0.0174	0.8958	0.0060	0.9390
N addition as KNO_3																
Vegetation (VT)	13.5036	**0.0009**	15.2768	**0.0005**	36.9175	**0.0000**	63.3260	**<0.0001**	4.6249	**0.0392**	97.1657	**0.0000**	200.3676	**<0.0001**	0.0471	0.8295
N addition (N)	161.6485	**<0.0001**	304.9585	**<0.0001**	79.8319	**0.0000**	39.5017	**<0.0001**	6.0634	**0.0194**	2.6717	0.1119	12.7754	**0.0011**	16.3227	**0.0003**
Glucose (Glu)	12.8603	**0.0011**	35.1288	**<0.0001**	124.7803	**0.0000**	10.1051	**0.0033**	0.0075	0.9315	2.5656	0.1190	47.6605	**<0.0001**	41.7231	**0.0000**
Freezing (F)	33.8852	**<0.0001**	31.5531	**<0.0001**	108.2297	**0.0000**	42.7954	**<0.0001**	160.9946	**<0.0001**	6.0254	**0.0197**	23.2876	**<0.0001**	55.0273	**0.0000**
VT × N	33.1961	**<0.0001**	3.1412	0.0859	5.3563	**0.0272**	24.3457	**<0.0001**	0.7460	0.3942	4.9697	**0.0329**	22.9096	**<0.0001**	3.8351	0.0590
VT × Glu	5.6421	**0.0237**	5.0713	**0.0313**	20.2666	**0.0001**	0.7701	0.3867	4.1056	0.0511	0.6340	0.4317	1.7496	0.1953	0.3653	0.5498
VT × F	0.9309	0.3419	0.8292	0.3693	18.7232	**0.0001**	7.3363	**0.0108**	28.0813	**<0.0001**	3.2761	0.0797	0.1591	0.6926	7.1654	**0.0116**
N × Glu	4.6014	**0.0396**	8.7088	**0.0059**	16.8007	**0.0003**	0.0259	0.8732	2.7132	0.1093	0.5446	0.4659	1.3946	0.2463	0.0010	0.9750
N × F	1.9264	0.1747	0.0743	0.7869	0.3860	0.5388	4.5612	**0.0405**	0.8776	0.3559	5.6073	**0.0241**	0.9219	0.3442	8.7711	**0.0057**
Glu × F	0.0202	0.8879	6.8524	**0.0134**	5.8177	**0.0218**	0.4916	0.4883	1.1887	0.2837	0.1359	0.7148	1.7725	0.1925	1.7286	0.1979
VT × N × Glu	0.0003	0.9856	0.0945	0.7606	0.2749	0.6037	3.9264	0.0562	0.2303	0.6346	0.1762	0.6774	3.6760	0.0642	3.5045	0.0704
VT × N × F	1.0175	0.3207	22.0623	**<0.0001**	5.3738	**0.0270**	19.9835	**0.0001**	3.0046	0.0927	0.0394	0.8440	11.8018	**0.0017**	12.8121	**0.0011**
VT × Glu × F	5.6401	**0.0237**	0.7776	0.3845	10.3349	**0.0030**	0.0002	0.9887	0.4666	0.4995	0.5498	0.4638	0.6971	0.4099	0.0123	0.9124
N × Glu × F	1.5977	0.2154	0.0005	0.9832	1.5542	0.2216	0.5426	0.4667	0.3612	0.5521	2.8255	0.1025	0.8872	0.3533	0.7591	0.3901
VT × N × Glu × F	0.2056	0.6533	0.2074	0.6519	2.6582	0.1128	0.1994	0.6582	1.0220	0.3196	0.9839	0.3287	0.0000	0.9951	1.1725	0.2870
